# Advances in Nanoplasmonic Biosensors: Optimizing Performance for Exosome Detection Applications

**DOI:** 10.3390/bios14060307

**Published:** 2024-06-14

**Authors:** Devi Taufiq Nurrohman, Nan-Fu Chiu, Yu-Sheng Hsiao, Yun-Ju Lai, Himansu Sekhar Nanda

**Affiliations:** 1 Laboratory of Nano-Photonics and Biosensors, Institute of Electro-Optical Engineering, National Taiwan Normal University, Taipei 11677, Taiwan; 81077004h@ntnu.edu.tw; 2 Department of Life Science, National Taiwan Normal University, Taipei 11677, Taiwan; yunjulai@ntnu.edu.tw; 3 Department of Materials Science and Engineering, National Taiwan University of Science and Technology, No. 43, Sec. 4, Keelung Road, Da-an District, Taipei 10607, Taiwan; yshsiao@mail.ntust.edu.tw; 4 Biomedical Engineering and Technology Laboratory, Mechanical Engineering Discipline, PDPM Indian Institute of Information Technology, Design & Manufacturing, Jabalpur 482005, India; himansu@iiitdmj.ac.in

**Keywords:** surface-plasmon resonance, localized surface-plasmon resonance, biosensors, exosome

## Abstract

The development of sensitive and specific exosome detection tools is essential because they are believed to provide specific information that is important for early detection, screening, diagnosis, and monitoring of cancer. Among the many detection tools, surface-plasmon resonance (SPR) biosensors are analytical devices that offer advantages in sensitivity and detection speed, thereby making the sample-analysis process faster and more accurate. In addition, the penetration depth of the SPR biosensor, which is <300 nm, is comparable to the size of the exosome, making the SPR biosensor ideal for use in exosome research. On the other hand, another type of nanoplasmonic sensor, namely a localized surface-plasmon resonance (LSPR) biosensor, has a shorter penetration depth of around 6 nm. Structural optimization through the addition of supporting layers and gap control between particles is needed to strengthen the surface-plasmon field. This paper summarizes the progress of the development of SPR and LSPR biosensors for detecting exosomes. Techniques in signal amplification from two sensors will be discussed. There are three main parts to this paper. The first two parts will focus on reviewing the working principles of each sensor and introducing several methods that can be used to isolate exosomes. This article will close by explaining the various sensor systems that have been developed and the optimizations carried out to obtain sensors with better performance. To illustrate the performance improvements in each sensor system discussed, the parameters highlighted include the detection limit, dynamic range, and sensitivity.

## 1. Introduction

Exosomes are the smallest material in extracellular vehicles. They have diameters of 30–150 nm, are produced by cells, and are released into the extra-cellular environment. This type of material is found in all biofluids, including blood, urine, saliva, synovial fluid, and cerebrospinal fluid [1,2]. Recent reports state that an exosome can contain 4563 proteins, 194 lipids, 1639 messenger ribonucleic acids (mRNAs), and 764 micro RNAs (miRNAs) [3,4]. Due to their abundant presence, exosomes have recently been considered as promising biomarkers in the early diagnosis of diseases because exosomes released by cells directly reflect the pathological condition of the host cells [2].

Due to the above-mentioned advantages, the analysis and quantification of exosomes has become one of the widely explored topics for the purpose of early detection of diseases. Several methods have been developed for this purpose, such as nanoparticle tracking analysis [5], flow cytometry [6], dynamic light scattering [7], and Western blot [8]. However, because the process is time consuming and the operation requires skilled operators, mass sample testing is difficult to realize. Currently, biosensors have become an alternative method for exosome quantification. Huang et al. developed a fluorescence aptasensor to detect gastric cancer exosomes [9]. The developed biosensor is composed of a Mucin 1 (MUC1)-specific aptamer as a probe and branched rolling circle amplification (BRCA) as an agent for signal amplification. SYBR Green I has been used as a fluorescence dye, and this sensor system has succeeded in detecting exosomes up to a concentration of 4.27 × 10^4^ exosomes/mL, with a linear response ranging from 10^5^ to 10^9^ exosomes/mL. The fluorescence biosensor has also been successfully used to detect different exosomes, such as the exosomes extracted from tumor cells [10,11], lung cancer [12], and breast cancer [13]. Other researchers, namely Pan et al., have developed a surface-enhanced Raman scattering (SERS) biosensor based on gold nanostars (AuNSs) decorated with MoS_2_ [14]. In this study, the ROX-labeled aptamer (ROX-Apt) was used as a probe to bind the target transmembrane protein CD63 (a representative surface marker on exosomes). The presence of exosomes will result in the release of the ROX-Apt probe from the nanocomposite surface, and this phenomenon is characterized by a decrease in the SERS signal. This SERS aptasensor can detect exosomes in a wide range from 55 to 5.5 × 10^5^ exosomes/μL, with a detection limit of 17 exosomes/μL. Currently, many biosensors have been integrated with microfluidic technology and artificial intelligence to obtain more accurate sensors for analyzing more complex samples [13,15,16,17].

In addition to the sensors discussed above, nanoplasmonic biosensors have become widely used sensors because they are easy to operate, label-free, and offer real-time detection. The label-free charactersistics of biosensors can reduce the experimental complexity that arises from the use of labels. For SPR-based biosensors, this type of sensor has a decay length that is comparable to the size of the exosome. Some researchers reported that the penetration depth of conventional SPR biosensors is <300 nm [18,19,20,21]. These properties make SPR biosensors ideal for use in exosome research [22,23]. On the other hand, the decay length of the LSPR biosensor has a shorter range, namely around 6 nm [18,24,25,26]. A decay length that is too short means that the surface-plasmon field cannot penetrate deeply enough into the analyte, and this may result in inconsistent sensor response and invalid results. To overcome this, it is necessary to expand the response area of the sensor, for example by using additional layers or optimizing the gaps between particles, which can strengthen the surface-plasmon field so that it can penetrate the analyte more deeply [27,28,29].

**Figure 1:**
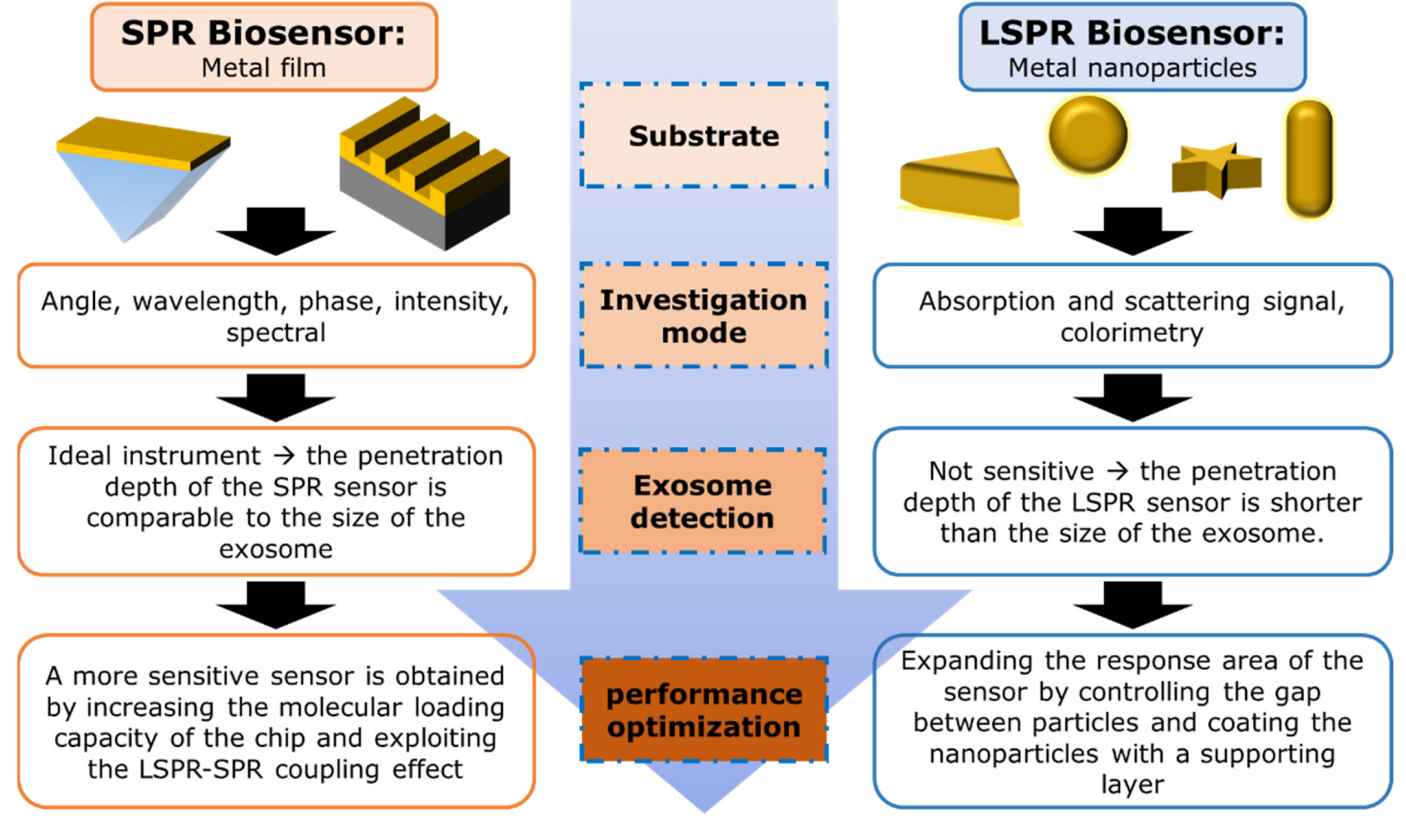
Diagram summarizing the foundations, investigation modes, and development directions of nanoplasmonic biosensors in the case of exosome detection.

This paper focuses on summarizing recent developments in SPR- and LSPR-based biosensors and their utilization for exosome detection. In simple terms, the contents of this paper are illustrated by the chart in Figure 1. For SPR-based biosensors, there are the two most popular approaches to improving their performance, namely by increasing the molecular loading capacity of the biosensor chip [30,31,32] or by exploiting the LSPR-SPR coupling effect [33]. For the LSPR biosensor itself, the analyzed signal can be obtained based on scattering spectra from dark field images or based on colorimetry. In the next section, we will briefly discuss the theoretical background of the SPR and LSPR phenomena. After explaining the potential of exosomes as biomarkers, we will summarize how SPR and LSPR signal amplification is performed and compare the resulting performance. The parameters highlighted in this context include the sensor’s detection limit, sensitivity, and its dynamic range.

## 2. Working Principles of Nanoplasmonic Biosensors

### 2.1. SPR-Based Biosensor

Surface plasmons are the collective oscillations of free electrons at the interface between a conductive material, such as a metal, and a dielectric or insulating material. Resonance in surface-plasmon waves occurs when the frequency of incident light matches the vibration frequency of electrons. In this situation, there is a transfer of energy from the incident light to the surface-plasmon wave. and this causes the intensity of the reflected light measured by the photodetector to decrease until it finally becomes zero after reaching the optimum resonance condition [34,35].

**Figure 2:**
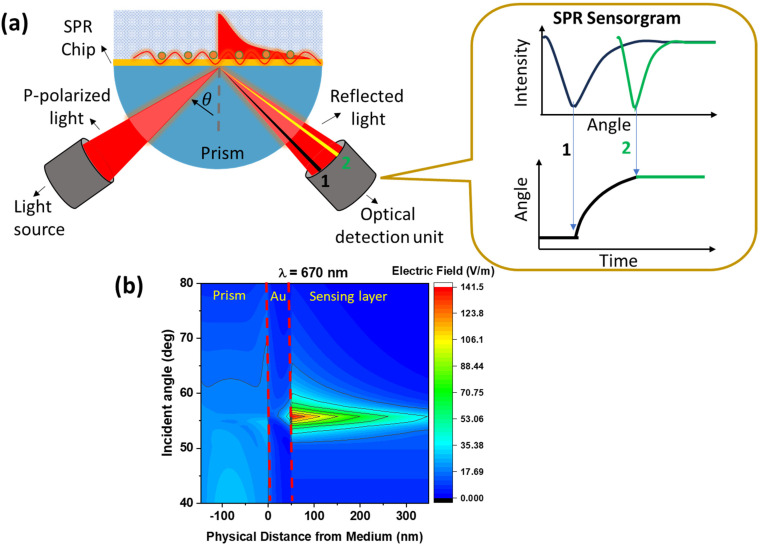
(**a**). Prism-coupled SPR biosensor with angle investigation mode. (**b**). Differences in electric field profiles at different incidence angles. Note: we added numbers 1 and 2 to (**a**) to explain how the sensorgram signal is acquired.

SPR biosensors cannot be excited directly by photons due to momentum mismatch. Coupling media is required to achieve matching conditions. Figure 2a below shows one type of SPR biosensor based on prism coupling with an incident-angle-based investigation mode. The phenomenon of binding and release on the sensing surface will cause a shift in the resonance angle, and this shift is monitored in real-time in the form of an SPR sensorgram. The higher refractive index of the analyte will cause the SPR angle to shift to a higher angle. Figure 2b shows how the magnitude of the electric field varies from different incident angles on an SPR chip composed of an SF10 prism as an optical component of the sensor and an Au film with a thickness of 50 nm. The dielectric medium is water. Computational results show that the resonance angle of this structure is around the incidence angle of ~56°. In resonance conditions, the electric field produced is very high, and this zone is an area that is very sensitive to changes in the refractive index [36,37,38]. From the contours displayed, there is a very significant difference in the magnitude of the electric field at the SPR angle compared to other angles.

**Figure 3:**
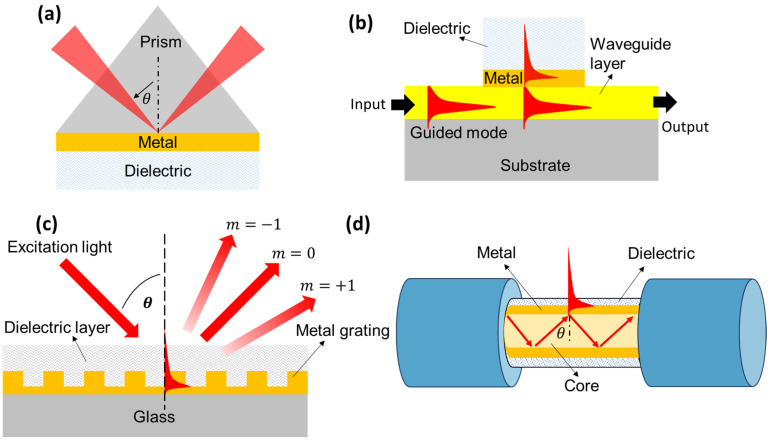
Various common configurations to achieve SPR. (**a**). Prism-based with Kretschmann configuration, (**b**). Waveguide based, (**c**). Grating based, (**d**). Optical fiber based.

The previous paragraph explains the SPR biosensor that uses a prism to implement it. Other coupling methods that can be used as alternatives include gratings [39,40], waveguide [41,42], and optical fiber [43,44]. The basic differences between these four coupling methods are shown in Figure 3. The SPR system coupled with a prism is a mature method [45,46]. In this sensor system, we can use the Otto or Kretschmann configuration. For Otto configuration, the thin layer of metal and the prism are separated by a gap with a thickness on the order of micrometers [47]. The performance of the sensor is highly influenced by the gap thickness, and due to the difficulty of precisely controlling the gap thickness, this configuration has not been widely explored in sensing applications. The second configuration is known as the Kretschmann configuration. As shown in Figure 3a, in this configuration, a thin layer of metal is deposited directly onto the surface of the prism. In general, prism-coupled SPR systems are expensive, and most of them are bulky in size. As a result, this sensor system is not suitable for long-distance measurements, thus limiting its practical application.

For SPR systems coupled with waveguides, the excitation method is like a prism-coupling configuration that utilizes total internal reflection [44,45,46]. As shown by Figure 3b, the prism is replaced by a waveguide, and when the guided mode transmitted from the input spreads to the sensing area that has been deposited with a metal film, the evanescent waves near the waveguide will penetrate the metal film. If the propagation constant of the guided mode matches the propagation constant of the surface-plasmon wave, resonance can occur.

Slightly different from SPR systems with prisms and waveguides, for SPR systems with gratings, light is emitted and directed towards the surface of the grating. The presence of a grating will divide the incoming light into several directions or diffraction orders (m). Please pay attention to the diffraction order in Figure 3c. Between these diffraction orders, there is a condition where the energy from the incident light moves to the metal surface to excite surface-plasmon waves, and this causes the intensity of the reflected light to decrease significantly. This condition can be achieved in both angular and wavelength investigation modes [48]. In addition to the above methods, fiber optic coupling has attracted the attention of researchers because of its compact structure, which allows it to be applied in narrow spaces, for real-time detection, and even in vivo in situ measurements. As shown in Figure 3d, the core of an optical fiber is coated with a thin layer of metal, and to produce SPR, three conditions must be met simultaneously [45]. First, some of the energy in the fiber core leaks into the fiber cladding. Second, the thickness of the metal layer should be moderate, generally 30–50 nm thick. Third, the polarization state of the cladding mode must be controlled. Various types of optical fiber have been successfully investigated, and some of them are polymer fiber, multi-core fiber, hollow fiber, and photonic crystal fiber [44].

There are several investigation modes that have been developed on SPR biosensors. Some of them are investigation modes based on angle, intensity, wavelength, phase, or a combination of angle and spectral [49,50]. We summarize related papers with different investigation modes in Table 1. It is also possible to combine the two coupling methods into an SPR sensor system. In this context, Cai et al. have integrated an SPR system with prism and grating; this integration is able to increase SPR sensitivity up to 2.81 times higher than conventional SPR sensors without grating [51]. Pandey et al. also integrated fiber optics and grating [52]. This sensor system has been successfully used to detect cortisol using both angular and intensity investigation modes.

Another development direction for SPR biosensors is in miniaturization and integration. Miniaturization and integration are carried out by creating tools that are smaller, portable, and can be integrated with other devices to obtain more complete information during experiments. Chiu et al. utilized electrochemical surface-plasmon resonance (EC-SPR) signals to quantitatively detect real-time changes in the removal of oxygen functional groups in electrochemically-reduced graphene oxide (ERGO) [69]. Miniaturization can also be done by integrating it with microfluidic technology. This integration offers the advantages of automation, small volumes, and fast processing, and can also increase the sensing efficiency of the sensor [70,71].

### 2.2. LSPR-Based Biosensor

**Figure 4:**
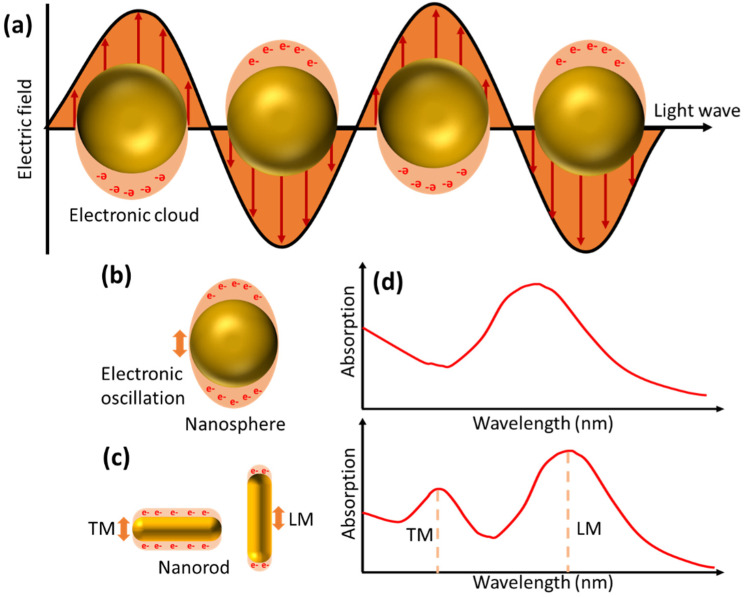
Schematic representation of (**a**) LSPR phenomena, electrical oscillations of (**b**) nanospheres, (**c**) nanorods, and (**d**) absorption spectra of nanospheres and nanorods.

In the previous section, we discussed the types of biosensors that utilize the propagation of surface-plasmon waves in their work. In this section, we will discuss plasmonic phenomena in a nanometal structure with a size much smaller than the wavelength of the incident light. When light interacts with this type of nanometal, the collective oscillations of electrons will be localized surrounding the nanoparticle [72]. This oscillation phenomenon is illustrated by Figure 4a. This occurs because the surface of the nanostructure is shorter than the propagating plasmon decay length, causing plasmon oscillations to tend to produce standing waves rather than propagating waves. The electromagnetic field brought by the incident light causes charge separation between the free electrons and the ionic metal nucleus. Furthermore, the repulsive force of coulomb repulsion between free electrons will act as a restoring force that causes collective oscillations of these electrons [24].

Materials that can be used as transducers in LSPR biosensors must meet the Frohlich conditions, namely [73]:

(1) $$\varepsilon_{r}=-{2\varepsilon }_{m}$$

In this context, $\varepsilon_{r}$ denotes the real part of the complex dielectric function of the nanoparticle, while $\varepsilon_{m}$ denotes the permittivity of the medium around the nanoparticle. From this equation, it can be concluded that, to implement an LSPR-based biosensor, we must use a material with a negative $\varepsilon_{r}$, and the imaginary part of the dielectric constant ($\varepsilon_{i}$) is expected to be positive or perhaps ignored. For this reason, Au and Ag are the types of metals that are widely used in LSPR biosensor applications due to their small imaginary dielectric values.

The LSPR phenomenon is characterized by very strong absorption bands at certain frequencies [74]. The LSPR signal can be tuned from the UV-visible to the infrared region by controlling the size, shape, and refractive index around the nanoparticle and its composition [75]. Figure 4b–d depicts oscillations in nanoparticles in the form of nanospheres and nanorods. Two different oscillation directions in the nanorod give rise to new absorption bands called transverse mode (TM) and longitudinal mode (LM). When the analyte binds to the nanoparticle surface, it will cause a change in the refractive index on the nanoparticle surface, which further shifts the LSPR peak frequency. The LPSR peak shift is also influenced by the arrangement and shape of the nanoparticles. Materials with greater negative real dielectric constants will produce sensors that are more sensitive to changes in the local refractive index [76]. In addition, nanoparticles with asymmetric shapes are more sensitive than spherical shapes. This type of nanoparticle shows higher field enhancement compared to conventional nanoparticles [77]. Jeon et al. compared the sensitivity of the LSPR biosensor on two different types of nanoparticles, namely gold nanospheres (AuNSs) and gold nanocubes (AuNCs) [78]. In this study, they found that AuNCs with sharp vertices and edges showed higher sensitivity compared to edgeless AuNSs of the same size. The field enhancement also provides advantages not only in the case of LSPR but can also be exploited to amplify surface-enhanced Raman scattering (SERS), surface-enhanced infrared absorption (SEIRA), and plasmon-enhanced fluorescence (PEF) signals.

## 3. Potential Use of Exosomes as Biomarkers

Cancer is a global health problem, especially in developing countries, causing millions of deaths every year [79]. This disease is characterized by the uncontrolled growth of abnormal cells that can spread to other parts of the body. One of the breakthroughs in cancer research is the use of exosomes as biomarkers and as a potential therapeutic tool [80]. In the case of therapeutics, exosomes can be modified with homing molecules via ligands, magnetic materials, charge affinity, and pH-responsive motifs for drug-delivery purposes [81]. Therapy can also be carried out using natural exosomes which have antitumor properties [82]. The development of exosome applications opens up new opportunities for more effective cancer diagnosis and treatment.

Although various diagnostic methods, such as computerized tomography (CT) for lung cancer, mammography and MRI for breast cancer, and colonoscopy for colorectal cancer, have been developed, several aspects still have problems that need to be resolved, such as high false-positive rates, low sensitivity, high costs, and uncomfortable procedures [83]. In addition, the accessibility and practicality of repeated examinations with these methods are also issues. Recently, the utilization and quantification of biomarkers have become the most promising approach in cancer diagnosis and therapy. Biomarkers, defined as “molecular signatures”, provide accurate information about the stage and mechanisms of cancer. Although many potential biomarkers have been identified, major gaps and challenges remain in implementing biomarker research into clinical practice. Some of the challenges include low concentrations of biomarkers in human body fluids and the poor stability of these potential biomarkers [84]. An example is the tumor necrosis factor (TNF-α) biomarker. In healthy humans, the concentration of this biomarker is typically ~20 pg/mL. The molecular weight of TNF-α is also lower than that of many common biomarkers (~17 kDa) [85,86]. Both of these properties are unfavorable and make detection of TNF-α still a significant challenge [87].

**Figure 5:**
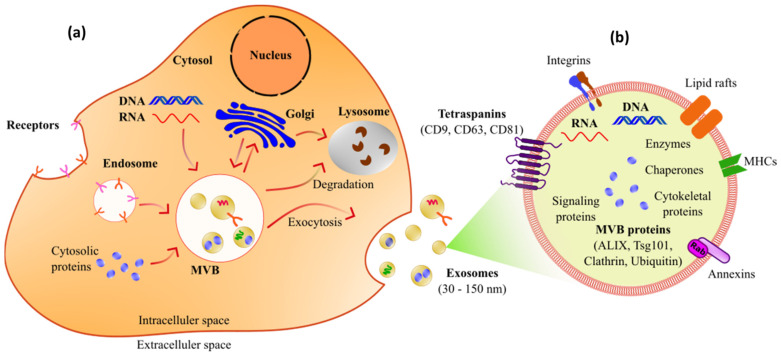
(**a**). Illustrates the process by which exosomes are produced and released from the cell and (**b**). shows the composition, including the surface markers of an exosome released from the cell.

Of the many potential biomarkers that have been identified, exosomes are one of the promising biomarkers. Exosomes are a type of small vesicle produced by various types of cells and have an important role in various biological processes, including intercellular communication, signal transmission, and the modulation of immune responses [88,89,90]. Figure 5a illustrates the process of exosome production and release from cells, and the overall composition, including exosome surface markers, is shown in Figure 5b. There are various types of biological materials in exosomes, including lipids, proteins, and nucleic acids [91]. Exosomes are defined by their mechanism of release into the extracellular environment via the fusion of late endosomes/multivesicular bodies (MVBs) with the plasma membrane [92,93]. In this case, the International Society for Extracellular Vesicles (ISEV) has determined the minimal experimental requirements to define exosomes with other extracellular vesicles (EVs) [94,95]. The biological materials contained in exosomes vary depending on physiological conditions and the external environment. The proteins contained in exosomes can be enzymes, growth factors, and membrane proteins. Lipids in exosomes mainly consist of phospholipids and cholesterol, which affect the stability and function of exosome membranes. In addition, exosomes also contain RNA, such as messenger RNA (mRNA), microRNA (miRNA), and other non-coding RNA, which can be transferred to recipient cells to influence genetic regulation and other biological processes. These rich properties make exosomes an important component in intercellular communication and a potential target for biomedical research and applications.

We note three main reasons that make exosomes a potential biomarker. First, exosomes are promising due to their high level of specificity. The diverse constituents of exosomes accurately reflect the cell’s origin and pathological conditions [96]. Moreover, the compositions of exosomes in different diseases may vary according to the disease progression and stage. Therefore, the biomarkers contained in exosomes are able to provide very specific information regarding the type and status of the disease being suffered. Second, the advantage of exosomes lies in their ease of access. As a heterogeneous part of EVs, exosomes are widely distributed in various body fluids, ranging from urine, blood, milk, saliva, cerebrospinal fluid, and amniotic fluid to sperm [97]. The ability to access exosomes from non-invasive samples, such as blood, will make sample collection easier, and this will make the process easier and more comfortable for patients. Third, the size of exosomes is significantly larger than that of biomolecules, and they have superior stability compared to other circulating biomarkers (i.e., proteins, nucleic acids, and metabolites) [98,99]. The lipid structure in the exosome membrane will provide protection for the molecules in the exosome from enzymatic degradation and external environmental factors. Exosome samples can be stored by freezing, freeze-drying, and spray-drying [100,101]. This stability makes exosomes an ideal choice for use in non-invasive or minimally invasive clinical applications, as well as in the development of biosensor systems that require stable samples.

## 4. Isolation of Exosomes

A problem that is often encountered in the isolation of exosomes from biological fluids is the presence of contaminants in the analyzed samples [102,103]. Therefore, to obtain exosomes with high purity, purification must be carried out strictly. If this aspect is not carried out, exosomes are usually contaminated by other membrane vesicles, such as shedding microvesicles (SMVs) and apoptotic blebs (ABs) [104]. There are at least five types of isolation techniques that have been developed, namely differential ultracentrifugation-based techniques, size-based techniques, immunoaffinity exosome capture-based techniques, and microfluidics-based techniques [105]. This technique utilizes the properties of exosomes at the isolation stage, such as density, shape, size, and proteins on the exosome surface. All of this will be explained in the following section.

**Figure 6:**
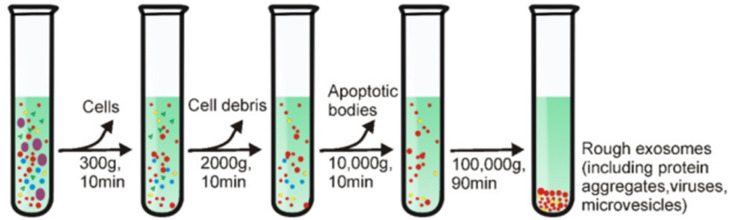
Schematic representation of differential ultracentrifugation-based exosome isolation [107]. Copyright (2020), Ivyspring International Publisher.

The first method is called differential ultracentrifugation. The working principle of the differential ultracentrifugation method utilizes differences in volume and physical properties of the material present in human tissue samples [106]. Figure 6 illustrates the exosome isolation steps using the density ultracentrifugation method [107]. The beginning step in this method is low-speed centrifugation (e.g., 300 g) to eliminate large particles such as cells. The centrifugation speed was then increased gradually to remove other contaminants in the sample, such as cell debris and apoptotic bodies. The exosomes that have been cleaned are then precipitated by centrifuging the sample at a speed of 100,000× *g* for 90 min. All these steps are carried out in an environment with a temperature of 4 °C.

**Figure 7:**
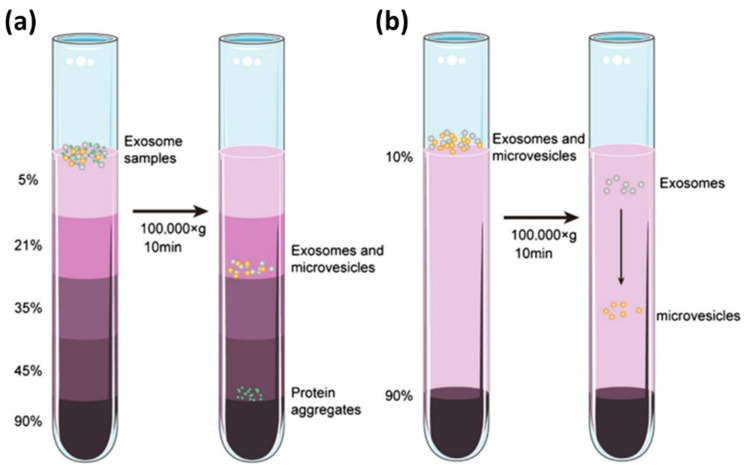
Schematic of gradient density ultracentrifugation-based exosome isolation. (**a**). Isopycnic density gradient ultracentrifugation. (**b**). Moving-zone gradient ultracentrifugation [108]. Copyright (2022), Springer Nature BV.

The second method for exosome separation is the density gradient method. The working principle of this method is carried out by utilizing the centrifugation force where, at a certain speed, the materials in the sample will settle in the isodensity zone. As shown in Figure 7, initially, the exosome sample is placed at the top of an inert gradient medium, such as a linear sucrose gradient (2.0–0.25 M sucrose) [106]. The gradient is then centrifuged at a certain speed and time at a temperature of 4 °C so that zones with different densities are produced in the sample. For exosomes, the density zone is in the range 1.10–1.18 g/mL. Because this method utilizes isodensity zones in exosome isolation, in several papers, this method is also called the zone centrifugation method [107].

**Figure 8:**
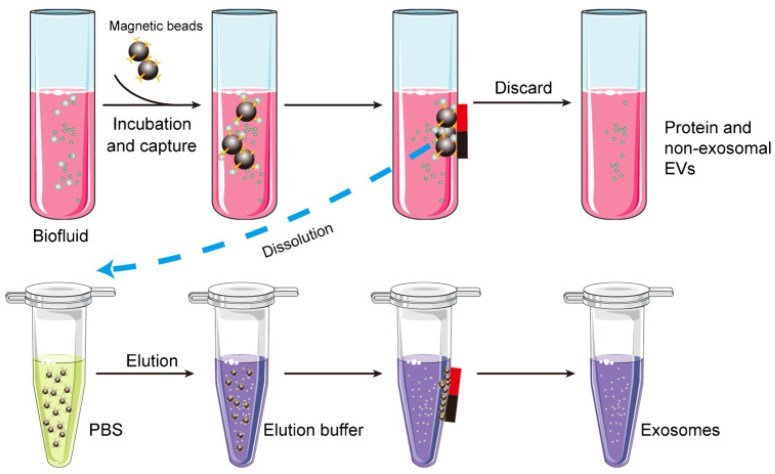
Schematic of the magnetic bead-based exosome isolation [108]. Copyright (2022), Springer Nature BV.

Exosomes are composed of proteins, lipids, and polysaccharides. Therefore, exosome isolation can be carried out by utilizing the bond between antigen and antibody. This technique is called the immunoaffinity technique, which is an approach that uses the affinity between antibodies and antigens to capture, purify, or detect certain compounds in a sample. As shown in Figure 8, antibodies on the surfaces of magnetic beads can be used to capture specific proteins, such as CD9, CD63, or CD81, on the surfaces of exosomes [108]. As a result, the exosomes obtained will have high purity. Apart from that, the use of magnetic material in this method can simplify the separation process and speed up the exosome isolation time.

Apart from the three methods discussed above, other alternative methods that can be used include size-exclusion chromatography (SEC), ultrafiltration, and precipitation. In SEC, exosomes are separated based on their molecular size as they pass through a column containing porous material [109]. Smaller molecules from the pores diffuse into the column pores, while larger molecules are blocked from exiting the pores and, then, are eluted from the column. SEC can isolate exosomes with high yield and purity, but this method is not suitable for processing large quantities of samples. In contrast to SEC, ultrafiltration utilizes a membrane with a certain pore size to separate exosomes from smaller particles. The sample is usually pressed or centrifuged through a membrane so that only small particles pass through, and the exosomes are retained. For precipitation, this method involves adding certain chemicals that cause exosomes to precipitate from the solution. Exosomes that have settled can then be separated via centrifugation. Exosome isolation is the first step in exosome research. Standard separation techniques are still challenging because they require large sample volumes, complex multi-step operations, are time-consuming, and require complex and expensive instruments. In contrast, microfluidic platforms have the potential to overcome some of these limitations, thanks to their high-precision processing, ability to handle fluids at the microscale, and their flexibility that can be integrated with various functional units, such as mixers, actuators, reactors, separators, and sensors, making these devices have a high potential for use in exosome isolation. We summarize the characteristics of each exosome isolation method in Table 2 below.

## 5. Development of SPR Biosensor for Exosome Detection

### 5.1. SPR Biosensor with Conventional Structure

SPR biosensors are analytical devices that have been used to monitor binding events between biomolecules, ranging from proteins, cells, exosomes, aptamers, peptides, lipids, and carbohydrates, to nucleic acids [119]. Compared with ELISA, which is the gold standard of immunoassay in clinical practice, SPR biosensors can provide fast and real-time affinity information and/or kinetic data. In addition, its characteristics of offering real-time monitoring, label-free detection, small sample size, and reusable sensor chips make it very reliable for quantifying biomolecules and understanding their interactions. This opinion is supported by research conducted by Hsu et al., where they compared the performance of the SPR biosensor with ELISA [120]. The chip used in this research is a conventional SPR chip, which is built from 2 nm Ti as the adhesion layer and a 49 nm Au film. To specifically detect tumor-derived exosomal proteins, the chip surface was functionalized with an anti-EGFR antibody using a PEG linker. This sensor system can be used to detect exosomes in a concentration range ranging from 5 × 10^9^ to 1.25 × 10^12^ exosomes/mL. When compared with ELISA, the SPR biosensor can be used to detect tumor-derived exosomal proteins up to a concentration of 3.5 × 10^9^ exosomes/mL. This value is 14 times lower than the detection limit of ELISA, which is only 5 × 10^10^ exosomes/mL.

**Figure 9:**
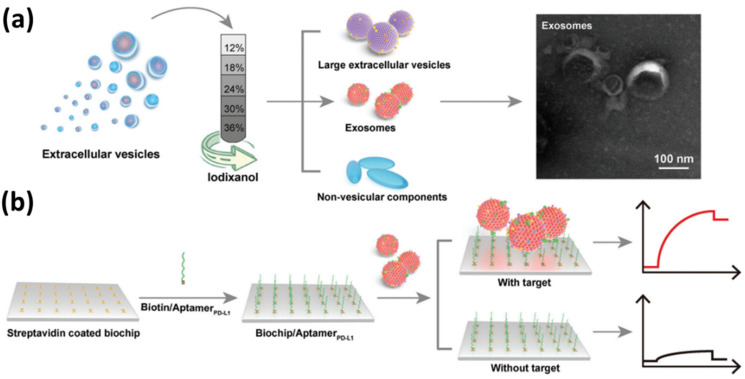
Overview of EVs separation and detection of exosomal PD-L1. (**a**) Schematic of isolation of extracellular vesicles (EVs) by iodixanol-based density gradient centrifugation method and obtained TEM images. (**b**) Functionalization of the SPR chip, detection of exosomal PD-L1 and illustration of the obtained SPR sensorgram signal. Note: The black and red lines in the SPR sensorgram indicate SPR signals without and with target exosomes, respectively [121]. Copyright (2022), The Royal Society of Chemistry.

Exosome detection using conventional SPR chips has also been carried out by Zhang et al. and Sina et al. Zhang et al. utilized an SPR biosensor to detect exosomal PD-L1 [121]. In this study, the detected exosomal PD-L1s were purified using the density gradient method. As shown in Figure 9a, crude exosomes were added to iodixanol dissolved in PBS at different concentrations. After the sample was centrifuged at 120,000× *g* at 4 °C overnight, the exosomes obtained were spherical or cup-shaped, with a size of 50–150 nm based on the results of TEM characterization. Furthermore, to be able to specifically detect exosomal PD-L1, the SPR chip was immobilized with PD-L1 aptamer by utilizing the bond between streptavidin and biotin (Figure 9b). In this study, the detection limit of the SPR sensor was 44.5 pM, with a linear range ranging from 0.63 nM to 7.5 nM.

In contrast to Zhang et al., Sina et al. used a conventional SPR chip to detect HER2 (+) breast cancer cells [122]. The SPR chip used is composed of 5 nm Ti and 50 nm Au and anti-HER2 antibodies are immobilized on the surface of the SPR chip by utilizing the bond between biotin and streptavidin. This sensor system can detect HER2 (+) exosomes up to a concentration of 0.828 × 10^4^ exosomes/μL, with a linear dynamic range ranging from 0.828 × 10^4^ to 3.31 × 10^4^ exosomes/μL.

A more comprehensive analysis in the investigation of exosomes was carried out by Zhang et al. [123]. They used conventional chips composed of 1 nm Cr and 47 nm Au films. The SPR biosensor developed is called plasmonic scattering microscopy (PSM), which integrates a prism-based SPR system with microscopy techniques. This device is designed to provide visual images of the exosomes being analyzed and determine their kinetic parameters. Kinetic parameters, such as association rate constants (k_on_), dissociation rate constants (k_off_), and equilibrium constants (K_D_) of binding of different ligands (anti-CD81 and anti-EGFR) and analytes (A431 and 293T exosomes), were successfully calculated. The results of this study show that the SPR biosensor with a conventional structure not only has good sensitivity for exosome detection but also provides more comprehensive information in analyzing exosomes.

### 5.2. SPR Biosensor Modified with 2D Material

With the rapid development of nanotechnology, nanomaterials have been utilized to obtain more sensitive SPR biosensors. In simple terms, SPR signal amplification can be achieved by utilizing two-dimensional (2D) materials and exploiting the LSPR coupling effect produced by metal nanoparticles, such as AuNPs [124,125,126] and AuNR [127]. The presence of 2D materials, such as graphene, MoS_2_, and Ta_2_C MXene, on the surfaces of SPR chips can effectively increase the surface electric field intensity and also surface active sites [128,129,130]. For graphene-based SPR biosensors, the increase in sensitivity is caused by the graphene plasmons coupling with surface-plasmon polaritons, resulting in the amplification of the evanescent field intensity and propagation length [131]. In contrast to the graphene-based SPR biosensor, the amplification of the electric field intensity in the MoS_2_-based SPR biosensor is caused by effective electron transfer initiated by the high work function of carboxyl MoS_2_ [132,133]. Increasing the electric field and plasmon propagation range on the sensing surface is one of the factors that causes SPR biosensors to become more sensitive.

Chen et al. immobilized multifunctional peptides (M-Pep, SS-IMVTESSDYSSY-KK-FHYQRDTPKSYN) on the surfaces of different SPR chips, namely a bare SPR chip a and SPR chip grown with graphene and MoS_2_ [134]. If the SPR response of these three chips is compared, the graphene-based SPR chip has the highest response, and this indicates a higher molecular loading capacity. The SPR angle shifts for bare SPR chip, and graphene and MoS_2_-based SPR chips are 24 m°, 100 m°, and 79 m°, respectively. This chip has been applied to detect PD-L1 exosomes, and the graphene-based sensor can detect exosomes up to a concentration of 20 exosomes/mL, with a dynamic range ranging from 10^4^ to 10^8^ exosomes/mL.

**Figure 10:**
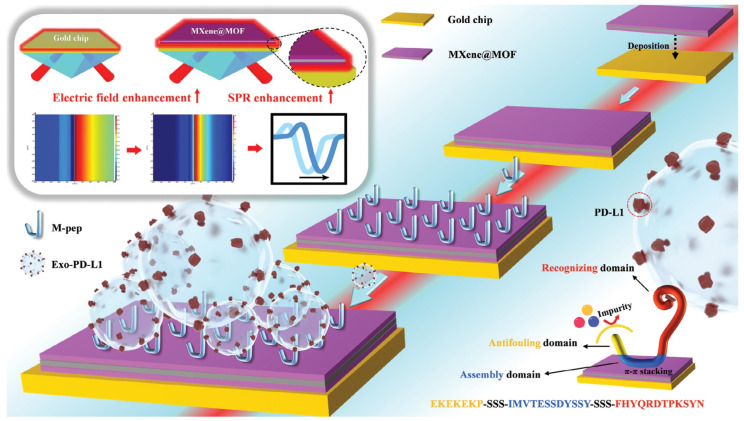
Construction process of MXene@MOF enhanced SPR sensor and illustration of the electric field enhancement phenomenon [135]. Copyright (2023), Small.

Other researchers, namely Wang et al., used a different type of 2D material, namely the MXene@MOF2D heterojunction [135]. In this study, the sensitivity of different SPR chips was also investigated. The result is that the SPR angle shift that occurs from chips modified by 2D material is always higher than that of bare gold chips. Interestingly, the MXene@MOF-modified gold chip has the highest signal response compared to the other three chips. The sensitivities of bare gold-based and MXene-, MOF- and MXene@MOF-based SPR chips are 73.07°/RIU, 132.97°/RIU, 131.53°/RIU, and 166.17°/RIU, respectively. In addition, the magnitude of the electric field in the sensing surface of the gold chip modified with MXene@MOF is the highest, which is about 117.6% higher than that of the bare gold chip. This chip has been applied to detect PD-L1 exosomes. As shown in Figure 10, the SPR chip is functionalized with peptides containing three structural domains: an antifouling structural domain (EKEKEKP), a self-assembly structural domain (IMVTESSDYSSY), and a recognition structural domain (FHYQRDTPKSYN). The results obtained show that the SPR chip modified with the MXene@MOF heterojunction can be used to detect PD-L1 exosomes up to a concentration of 5.24 exosomes/mL. This value is 6.24 times lower than conventional SPR chips, where the detection limit is only 32.71 exosomes/mL.

The investigation mode of the two papers discussed previously uses the angle investigation mode; a different investigation mode has been developed by Hedhly et al. [136]. In this study, they have detected cancer-derived exosomes by utilizing the Goos–Hanchen signal. The chip that was developed is made from 40 nm Au film, which is deposited with a thin layer of 2D Ge_2_Sb_2_Te_5_ (GST). The thickness of the GST layer is optimized to achieve a zero-reflection state, which will result in a very sharp phase change at the resonance angle. To assess the performance of the biosensor, the biosensor based on Goos–Hanchen shift measurements has been compared with the conventional SPR sensing technique. Experimental results show that the developed biosensor has a detection limit of up to 10^4^ exosomes/mL, which is more than two orders of magnitude superior to conventional SPR sensing techniques. Different types of 2D materials and their utilization to increase the sensitivity of SPR biosensors have been discussed. Currently, other types of 2D materials have also been successfully used, such as 2D MOF Cu-TCPP by Wang et al. in 2022 and single-walled carbon nanowires by Zhou et al. in 2024 [137,138]. The resulting performance after chip modification is briefly summarized in Table 3.

### 5.3. SPR Biosensor Modified with Metal Nanoparticles

**Figure 11:**
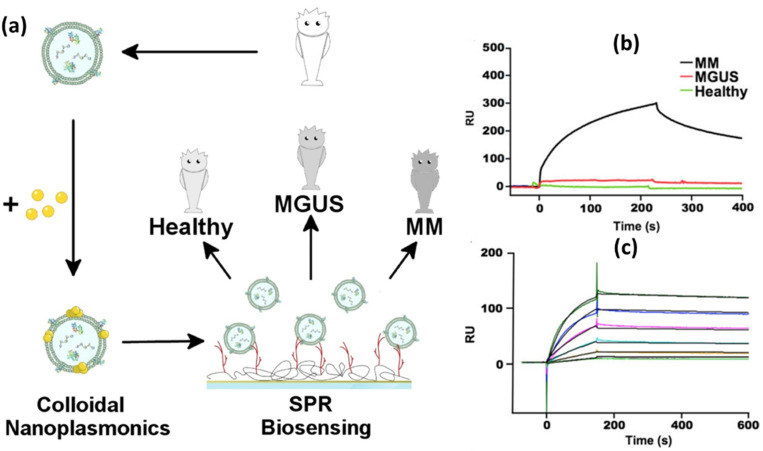
(**a**). Detection mechanism of the biosensor (**b**). SPR sensorgrams for exosomes (all at 4.4 nM) from healthy individuals or from MM and MGUS patients. (**c**). SPR sensorgrams for MM exosomes at decreasing concentrations (2.2, 1.1, 0.55, 0.32, 0.13, and 0.06 nM, from top to bottom) [139]. Copyright (2015), Elsevier B.V.

In the previous section, we discussed SPR signal amplification by utilizing 2D materials; now we will discuss the second signal amplification method, which is by utilizing the LSPR coupling effect. In this context, Noto et al., in 2016, amplified the SPR signal by utilizing AuNPs with a diameter of 14 nm to detect multiple myeloma (MM) [139]. The investigation began with the purification of exosomes using a sucrose-based density gradient method. The pure exosomes were then incubated in an Au NPs solution with a concentration of 6 nM so that Au NPs clusters would form on the exosome membrane as shown in Figure 11a. Success at this stage is marked by a red shift in the LSPR signal obtained from UV-Vis characterization. MGUS is a monoclonal gammopathy of undetermined significance and is an early phase of MM. In this condition, clonal plasma cells grow without causing clear and significant clinical symptoms. Figure 11b shows that the developed sensor system can be utilized for profiling MM-derived exosomes. Three different samples which are healthy, MGUS and MM have been measured and from the resulting SPR sensorgram, there is a quite clear difference in sensor response between the healthy and MGUS samples. Different concentrations of MM have also been measured up to a concentration of 0.06 nM. From the experimental results shown in Figure 11c, they justified that the detection limit of the sensor developed was 10 pM. Using the same amplification method, Liu et al. succeeded in detecting circulating exosomes (crEVs) by utilizing the natural receptor Tenascin-C (TNC). AuNPs that have been coated with cancer cell membranes are immobilized on the surface of the SPR chip, and by utilizing the specific interaction that occurs between TNC on the cancer cell membrane and fibronectin 1 (FN1) on crEVs, this sensor system is able to detect circulating exosomes up to a concentration of 18.1 exosomes/mL, with a linear range from 3 × 10^4^ to 3 × 10^7^ exosomes/mL [140].

**Figure 12:**
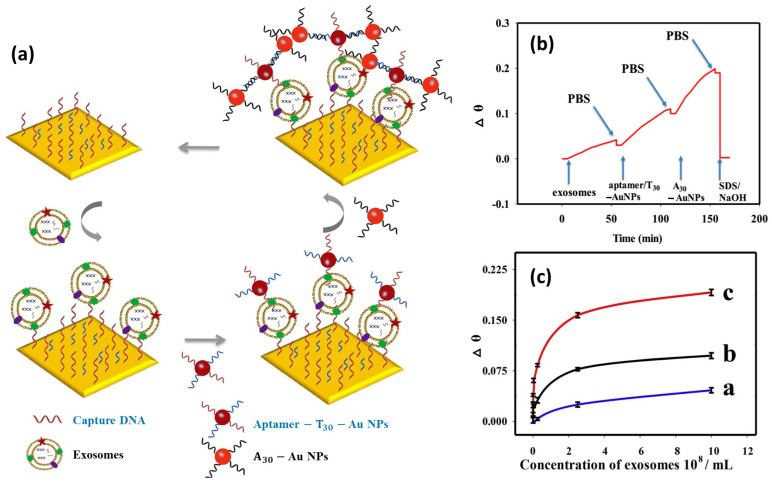
(**a**). Dual Au NPs-assisted signal amplification for exosomes detection. (**b**). SPR response in situ of the dual Au NPs-assisted SPR sensor. (**c**). The relationship between Δθ and exosome concentration using different sensing strategies. Note: Labels a, b and c in Figure 12c indicate signals with direct measurement, single AuNPs amplified SPR aptasensor and dual AuNPs amplified SPR aptasensor, respectively [141]. Copyright (2019), Elsevier B.V.

Amplification of the SPR signal with a more complex system, namely dual amplification, was carried out by Wang et al. [141]. In this study, they compared the performance of the SPR biosensor in the case of detecting cancer-cell exosomes using different detection strategies, namely direct measurement, and single and dual amplification using Au NPs. Two AuNP samples were functionalized with aptamer T_30_ and A_30_ to produce new nanoparticles called Aptamer-T_30_-AuNP and A_30_-AuNP. As shown in Figure 12a, to implement the dual-amplified SPR biosensor, Aptamer-T_30_-AuNP was designed to capture A_30_-AuNP via the hybridization of two complementary sequences (T_30_ and A_30_). The SPR signal obtained after dual amplification is shown in Figure 12b. If we compare the SPR angle shifts (Δθ) of the three types of measurements shown in Figure 12c, the dual-amplified SPR signal shows a higher response compared to the other two measurement methods. The results of this study show that the dual-amplified SPR biosensor has a detection limit that is 20 times lower than single amplification. The detection limits of the SPR biosensor with direct detection, single amplification, and dual amplification were 6.5 × 10^7^ exosomes/mL, 1.0 × 10^5^ exosomes/mL, and 5.0 × 10^3^ exosomes/mL, respectively.

**Figure 13:**
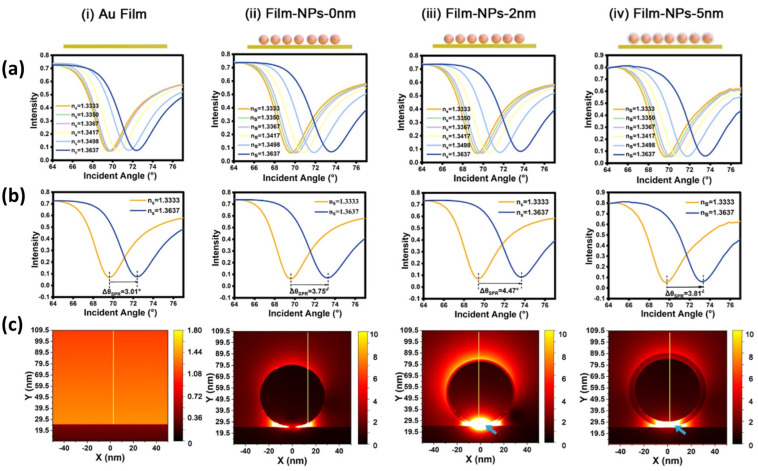
Original SPR spectra of different designed sensing interfaces while flowing through solutions with different refractive index (RI) (**a**,**b**) the resonance angle deviations for a given RI change. The electric fields at the designed different interfaces simulated by the FDTD simulation (**c**) [142]. Copyright (2023) American Chemical Society.

The effect of gap distance on enhancing sensitivity has been investigated by Zhou et al. [142]. As shown in Figure 13a, there are four structures investigated in this study, namely bare Au film and Au NPs on the Au film surface with different gap distances (0 nm, 2 nm, and 5 nm). Gap distances of 2 and 5 nm were achieved by coating the AuNP’s surface with SiO_2_. As shown by the SPR curve in Figure 13b, the presence of a gap greatly influences the SPR signal response. The higher the gap distance, the higher the SPR angle shift occurs. However, when the gap distance is increased to 5 nm, the SPR signal response decreases from 4.47° to 3.81°. However, this shift is still higher than that of bare Au film and SPR chips with a gap distance of 0 nm. To understand the mechanism behind the SPR enhancement, FDTD simulations are used to investigate the electric field at various designed interfaces. The result is that the electric field intensity at the 2 nm gap is 100 times greater than that of the bare Au film (Figure 13c). Even with a gap of 5 nm, the electric field intensity remains greater than that of the bare Au film; this result agrees with the results shown in Figure 13b. This biosensor system has been applied to detect PD-L1 exosomes, and the results are that this sensor system can be used to detect PD-L1 exosomes from a concentration of 10 exosomes/mL to 5 × $10^{3}$ exosomes/mL. Enhancing sensitivity by utilizing gap has also been carried out by Mao et al. using a different type of nanoparticle, namely AuNR [143]. To obtain a stable substrate, 2D MOF (Cu-Tcpp) was immobilized on the surface of the SPR chip, and DNA tetrahedrons were utilized to create a controlled gap between the gold thin layer and the AuNR. The experimental results show a significant increase, where the presence of AuNR can increase the electric field intensity by almost four times the previous condition. Sensor sensitivity also experienced a marked improvement, increasing from 127°/RIU on the chip without modification to 253.31°/RIU on the chip with gap mode.

SPR biosensors with conventional chips and modified chips have been discussed. Several approaches, modifications, and integrations have been carried out to obtain a more sensitive SPR biosensor. For more complex SPR chips such as pyramid-shaped nanohole-based plasmonic metastructures have been developed by Liang et al. to detect prostate cancer [144]. We summarize the utilization of SPR technology in both conventional and modified chips in the case of exosome detection in Table 3. In this table, the developed sensor system, recognition elements, targets, and resulting detection limits are the information highlighted in each paper reviewed. Due to the difference in units, we performed a unit conversion to compare the performance of each sensor. Because some data are not available in the article, we use several assumptions, and to differentiate them from the original detection-limit values, we denote them both with ${LOD}_{ori}$, which is the original detection-limit value, and ${LOD}_{conv}$, which shows the converted detection-limit value.

Because most of the units used are exosomes/mL, other units will be converted to this unit. To convert from molar (mol/L) to particles/mL, we use the Avogadro number. Therefore, 44.5 pM and 0.06 nM after conversion will change to 2.68 × 10^10^ exosomes/mL and 3.61 × 10^10^ exosomes/mL. Next, to convert from μg/mL to exosomes/mL, the molecular weight of the exosomes must be determined. Exosomes have a diameter between 30 and 150 nm. At this size, exosomes consist of hundreds to thousands of individual molecules. If we take the average, the molecular weight of exosomes will have a very wide range. The molecular weight of exosomes ranges from ~10 to 1200 MDa [145,146]. Because information regarding molecular weight was not provided by the authors of the article, the molecular weight of the exosomes was assumed to be 500 MDa. Therefore, the ${LOD}_{conv}$ for exosomes with a concentration of 2.75 × 10^−3^ µg/mL is 1.99 × 10^27^ exosomes/mL. From the ${LOD}_{conv}$ data below, for conventional SPR sensors, the smallest detection limit is only 8.28 × 10^6^ exosomes/mL. By modifying the chip using either 2D materials or metal nanoparticles, this method has been proven to be able to reduce the detection-limit value of the sensor.

**Table 3:** Summary of SPR sensors for the detection of exosomes.

Recognition Element	Specific Target	Developed Biosensor System	Detection Limit		Ref.
			${LOD}_{ori}$	${LOD}_{conv}$ (Exosomes/mL)	
**Conventional SPR Biosensors**					
Aptamer	PD-L1 exosomes	Exosome detection was carried out by utilizing the interaction of streptavidin and biotin using a conventional SPR chip	44.50 pM	2.68 × 10^10^	[121]
Anti-HER2	HER2 (+) Exosome	Conventional SPR chip was functionalized with anti-HER2	0.828 × 10^4^ exosomes/μL	8.28 × 10^6^	[122]
anti-EGFR	EGFR exosomes	Conventional SPR chip was functionalized with anti-EGFR	3.5 × 10^9^ exosomes/mL	3.5 × 10^9^	[120]
Biotinylated antibody	EGFR variant-III	SPR Chip based on Titanium nitride (TiN)	2.75 × 10^−3^ µg/mL	1.99 × 10^27^	[147]
**SPR Biosensors Modified with 2D Materials**					
Peptide	PD-L1 exosomes	Gold-based SPR chips deposited with graphene	20 exosomes/mL	20	[134]
peptide	PD-L1 exosomes	Sensitivity-enhanced SPR biosensor with MXene@MOF heterostructure	5.24 exosomes/mL	5.24	[135]
Peptide	PD-L1 exosomes	SPR chip was deposited with a 2D metal–organic framework (MOF)	16.7 exosomes/mL	16.7	[137]
peptide	PD-L1 exosomes	Enhancing the sensitivity of the SPR biosensor is carried out by utilizing the large surface area properties of single-walled carbon nanowires	75.23 exosomes/mL	75.23	[138]
Antibody	Anti-CD81	The sensitivity of the Goos–Hanchen (GH) shift-based SPR biosensor is enhanced with a thin layer of Ge_2_Sb_2_Te_5_ (GST)	10^4^ exosomes/mL	10^4^	[136]
**SPR Biosensors Modified with Metal Nanoparticles**					
Heparin	multiple myeloma	The SPR signal was amplified with Au NPs	0.06 nM	3.61 × 10^10^	[139]
Aptamer	hepatic carcinoma SMMC-7721	The SPR signal was amplified using AuNPs coated with polydopamine	5.6 × 10^5^ exosomes/mL	5.6 × 10^5^	[148]
molecular aptamer beacon (MAB)	HER2-positive exosomes	The SPR signal was amplified with AuNPs coated with tyramine	1 × 10^4^ exosomes/mL	10^4^	[149]
DNA	MCF-7 breast cancer cells	SPR biosensor with dual AuNP-assisted signal amplification	5 × 10^3^ exosomes/mL	5 × 10^3^	[141]
aptamer-DNA linker	LNCaP	SPRi with signal amplification with hydrogel-AuNP supramolecular sphere	1 × 10^5^ exosomes/mL	10^5^	[150]
peptide	PD-L1 exosomes	Enhanced SPR sensitivity is due to the substantial increase in the electromagnetic field generated at the tips of the gold nanoflowers with multi-tip tiny petals	4.95 exosomes/mL	4.95	[151]

## 6. Development of LSPR Biosensor for Exosome Detection

In the introduction, we stated that the decay length of the LSPR sensor is only on the order of 6 nm. This value is much smaller compared to the size of exosomes. The LSPR sensor can only reach a small part of the exosome, and this means that the LSPR sensor response does not come from the whole exosome but rather a small part of it. The surface-plasmon field must be strengthened to expand the response area of the sensor. In this way, the field can penetrate the exosomes more deeply, and as a result, the measured signal becomes more valid.

**Figure 14:**
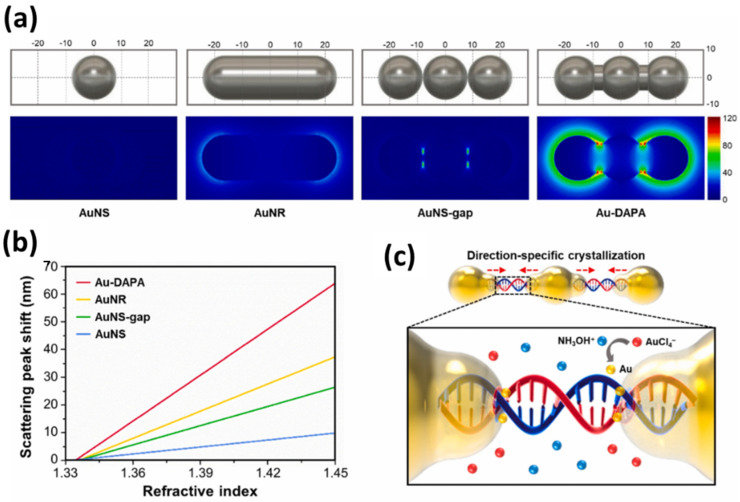
(**a**) Electric field profiles of LSPR biosensors with different structures and geometries (**b**) comparison of the sensitivities of the four types of nanoparticles investigated (**c**) Schematic diagram representing the crystallization process of Au atoms using a specific directional crystallization technique [27]. Copyright (2022), Elsevier B.V.

In the introduction, we stated that there are two approaches that can be taken to expand the response area of the LSPR sensor. Both are by coating metal nanoparticles with a supporting layer or by optimizing by controlling the gap between particles. Song et al. developed a DNA-assembled advanced plasmonic architecture (DAPA) structure to detect exosomal miRNAs (exo-miRNA) [27]. In this research, the distance between AuNPs was controlled at 2 nm using DNA, and they carried out computational studies using the finite-difference time-domain (FDTD) method to determine the electric field profile of each structure investigated. To assess the quality of the sensor, they compared the electric field and sensitivity of the DAPA structure with three other structures, namely spherical gold-shaped (AuNS), rod-shaped (AuNR), and rod-shaped with gaps (AuNS-Gap). From the investigation results shown in Figure 14a, there is a very significant difference in the electric field between the DAPA structure and the other three structures. A very strong electric field distribution is generated across the Au-DAPA surface caused by the presence of nanogaps that generate “hot-spots” that amplify the electromagnetic field energy by a higher enhancement factor. From the data shown in Figure 14b, the Au-DAPA structure also shows better sensitivity which is 1.66 times higher than that of the AuNR structure. To verify the computational results, Song et al. have succeeded in proving it experimentally [27]. The DAPA structure was obtained using a specific direction crystallization technique utilizing a hybridization process of two types of ssDNA-AuNP (1ssDNA-AuNP and 2ssDNA-AuNP). As shown in Figure 14c, the Au crystallization process is carried out by reducing ${{AuCl}_{4}}^{-}$ using ${{NH}_{3}OH}^{+}$. The detection method used is based on changes in Rayleigh light scattering of a single DAPA obtained from dark field microscope images. Of the three types of exo-miRNA, the detection limit of the sensor was 10.54 aM for exo-miR-125b, 13.53 aM for exo-miR-15a, and 11.10 aM for exo-miR-361.

**Figure 15:**
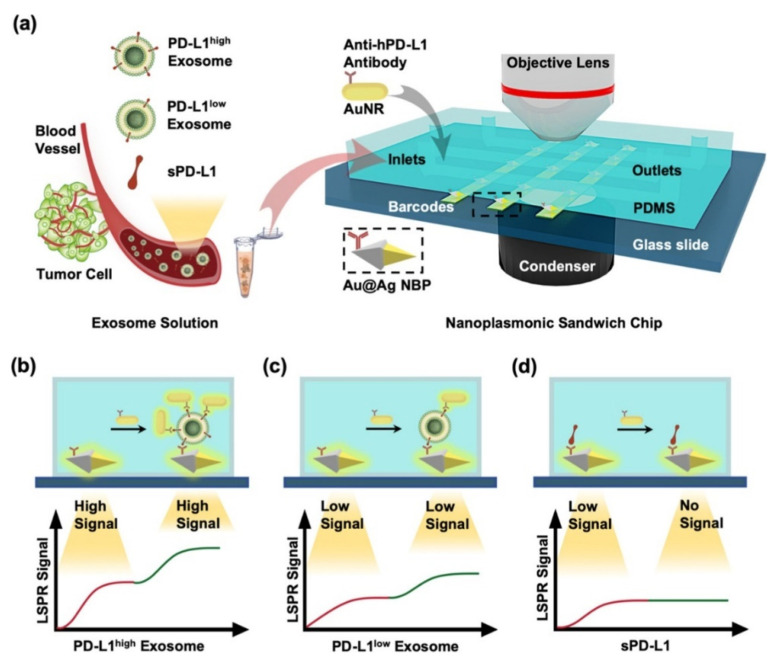
Sensing principle of the nanoplasmonic sandwich immunoassay for exosome quantification and subclass identification. (**a**) Detection procedure of the nanoplasmonic sandwich immunoassay. (**b**–**d**) Quantification and subclass identification of exosomes based on the generated primary (red curves) and secondary (green curves) LSPR signals [16]. Copyright (2021) American Chemical Society.

Apart from controlling the interparticle gap, the electric field in the LSPR biosensor can also be achieved by building a sensor system with a sandwich structure. Wang et al. developed a nanoplasmonic sandwich-based LSPR biosensor composed of Au@Ag core-shell nanobipyramid (NBP) and AuNR [16]. In this study, the electromagnetic field profile and LSPR scattering spectrum of different sensor systems, namely single NBP nanostructures, Au@Ag NBP exosome nanostructures, and Au@Ag NBP−exosome−AuNR sandwich nanostructures, were obtained from computational results using COMSOL Multiphysics software. The formation of Au@Ag NBP−exosome−AuNR sandwich nanostructures has resulted in an increase in shoulder-like peaks at a wavelength of 664 nm. At this wavelength, the electric field around the AuNR has a higher magnitude, and this is caused by the longitudinal plasmon band of AuNR. This sensor system has been utilized to detect PD-L1 exosomes. The detection mechanism in this study is summarized in Figure 15a. The glass slide was deposited and patterned with Au@Ag NBP to obtain a barcode based on Au@Ag NBP. This barcode is then functionalized with anti-hPD-L1 to specifically capture the exosomal PD-L1 or sPD-L1 present in samples injected via the microfluidic inlet. The bond that occurs between exosomes/sPD-L1 on the Au@Ag surface will result in a shift in the resonance wavelength, and the scattering intensity will increase due to changes in the local refractive index. Changes in this optical signal are monitored in real time via dark field images. After that, the secondary labeling agent, namely AuNR, was functionalized, as well as the anti-hPD-L1 antibody (anti-hPD-L1−AuNR). This sample is then injected and will be bound by PD-L1 exosomes so that a sandwich structure will be formed. As shown in Figure 15b–d, the measured signal is highly dependent on the amount of AuNR bound to the exosomes on the sensing surface. On the other hand, AuNRs will show strong LSPR scattering under dark field imaging with a different resonance wavelength (~660 nm) compared to Au@Ag NBPs (~720 nm). Due to this fact, identification and quantification of exosomal PD-L1 can be performed based on secondary LSPR signal intensity. With this detection mechanism, the LSPR biosensor is able to detect PD-L1 exosomes up to a concentration of 1.2 × 10^3^ exosomes/μL.

**Figure 16:**
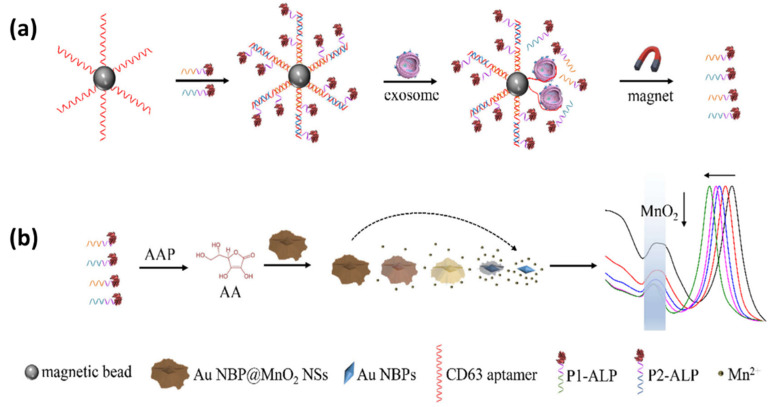
Schematic illustration of the plasmonic colorimetry for exosome detection via competitive reaction and etching of Au NBP@MnO_2_ NSs (**a**) Functionalization of magnetic beads (**b**) Detection mechanism of the LSPR sensor and illustration of the resulting signal [156]. Copyright (2023) American Chemical Society.

The two references discussed previously utilize the LSPR scattering signals obtained from dark field images to detect exosomes. Another approach, namely colorimetric sensing based on nanoplasmonic materials, can also be used as an alternative for detecting exosomes because of their abundant color variations. There are two methods of signal formation in plasmonic colorimetric sensing, namely (1) interparticle distance-dependent colorimetric assay based on target-induced forming cross-linking assembly/aggregate of plasmonic nanoparticles and (2) size/morphology-dependent colorimetric assay by target-controlled growth/etching of the plasmonic nanoparticles [152,153,154,155]. In the case of exosome detection, plasmonic colorimetric sensing was developed by Zhang et al. in 2022 [156]. In this study, they utilized the competitive reaction triggered by exosomes and etched the gold nanosheet structure nanobipyramid@MnO_2_ (Au NBP@ MnO_2_ NSs). As shown in Figure 16a, initially, the magnetic beads were functionalized with the aptamers CD63, P1-ALP, and P2-ALP. Furthermore, the presence of detected exosomes will break the hybridization complex (CD63/P1-ALP/P2-ALP aptamer) because the binding energy between the CD63 aptamer and CD63 has a stronger bond than the CD63 aptamer with P1-ALP and P2-ALP. The released P1-ALP and P2-ALP will catalyze the dephosphorylation of AAP to produce ascorbic acid (AA). The AA produced as a reductant etched the MnO_2_ nanosheets to produce Mn^2+^ and was oxidized to dehydrogenated ascorbic acid (DHA). The change in Au morphology caused by etching contributes to the blue shift in the LSPR band of Au NBP as shown by Figure 16b and also changes the visual color of the sample. Therefore, exosomes can be measured based on ALP-induced Au etching. With this approach, they confirmed the ability to detect exosomes up to a concentration of 1.35 × 10^2^ exosomes/μL.

To date, several researchers have succeeded in utilizing LSPR biosensors to detect exosomes. Li et al. used an optical microfiber-based LSPR biosensor to detect clear-cell renal cancer exosomes. Using the same assumptions as in the case of the SPR biosensor, we converted the detection-limit value into exosomes/mL unit to compare sensor performance. We summarize the results of the LSPR biosensor research for exosome detection in Table 4.

## 7. Conclusions and Future Perspective

In this paper, we have summarized recent developments in exosome detection using SPR- and LSPR-based biosensors. For SPR-based biosensors, we classify the structure of SPR biosensors based on their chips into conventional and modified SPR chips. The experimental results also show that the SPR biosensor with a conventional chip has reliable performance, where the detection limit of the SPR sensor is 14 times lower than ELISA. For early-detection purposes, SPR chip modifications are generally carried out to obtain a more sensitive sensor. In this case, two types of SPR signal amplification can be chosen, namely by utilizing 2D materials or by strengthening the plasmon coupling effect.

A number of researchers have successfully integrated SPR biosensors with microfluidic technology to detect different analytes [71,161,162]. However, it seems that this has not been done in exosome detection. In the case of exosome detection, the integration of microfluidic technology and SPR biosensors can provide significant advantages. The use of microfluidics can facilitate the isolation, purification, and separation of exosomes from complex samples, such as blood or other body fluids, thereby increasing the specificity of detection. Additionally, the integration of microfluidics with SPR biosensors can enable multiparameter analysis, in which multiple exosome components, such as proteins, lipids, and nucleic acids, can be detected simultaneously. This can provide more complete information regarding the characteristics of exosomes and their potential in disease diagnosis or biomedical research. The integration of microfluidic technology with SPR biosensors can increase the speed of analysis, enabling rapid and real-time detection of exosomes, which is critical for diagnostic applications that require rapid and accurate responses.

Compared with SPR-based biosensors, LSPR biosensors have not been widely explored in exosome detection. This is because the penetration depth is too short and not comparable to the size of the exosomes. The sensor chip must be modified by adding a supporting layer and controlling the gap between particles to strengthen the surface-plasmon field. LSPR chips are also often fabricated involving a combination of nanofabrication techniques [163]. Controlling the orientation of immobilized nanostructures is essential to ensure well-defined spectral signatures. To improve measurement reproducibility, the presence of a thin dielectric layer over the nanoplasmonic sensing array is another advantageous step to provide physical stability and also opens the door to various surface functionalization possibilities.

## Figures and Tables

**Table 1:** Comparison of performance of SPR sensors based on different optical substrates.

Optical Chip for SPR Sensor	Chip Used	Investigation Modes	Sensitivity	Detection Limit	Ref.
Prism	Conventional SPR chips based on gold	Angle	1.9 × 10^6^°/M	4.1 nM	[53]
	Conventional SPR chips based on gold	Angle	141.1°/RIU	-	[51]
	Conventional SPR chips based on gold	Phase	-	14.02 ng/mL	[54]
	SPR biosensor with signal amplified using Hybridization Chain Reaction	Phase	-	7.5 × 10^−7^ RIU	[55]
	Conventional SPR chips based on gold	Intensity	-	-	[56]
	SPR biosensor based on Au-Ag alloy film	Wavelength	5676.9 nm/RIU	-	[57]
	Near-infrared SPR sensor based on graphene-AuNPs architecture	Wavelength	39,160 nm/RIU	7.2 fg/mL	[58]
	Conventional SPR chips based on gold	Wavelength	1032 nm/RIU	-	[59]
Grating	Ag-based grating	Angle	128.85°/RIU	-	[60]
	Enhancement of the SPR sensitivity with Ag-Au bimetallic grating	Angle	346°/RIU	-	[61]
	Enhancement of the SPR sensitivity with Au-Al bimetallic grating	Angle	245.2°/RIU	-	[62]
	Au nanograting on silicon substrate	Wavelength	751 nm/RIU	23.5 nM	[63]
	Periodically corrugated gold film is coated with a thin antifouling polymer layer	Wavelength	-	1.1 nM	[64]
Waveguide	SiC waveguide-based SPR sensor is deposited with an Au-Ag bimetallic layer	Wavelength	2581 nm/RIU	-	[65]
	Polymer waveguide-based SPR sensor	Wavelength	4518 nm/RIU	2.2 × 10^−7^ RIU	[41]
	Dual channel planar waveguide-based SPR sensor	Wavelength	1500 nm/RIU.	-	[66]
Optical fiber	SPR fiber optic biosensor enhanced in sensitivity with graphene oxide	Wavelength	2471 nm/RIU	55 μM	[67]
	SPR fiber optic biosensor based on Au	Wavelength	1699 nm/RIU	-	[67]
	SPR biosensor based on tapered fiber optics	Wavelength	2100 nm/RIU	2.4 × 10^−10^ M	[68]

**Table 2:** The characteristics of each method in exosome isolation.

Isolation Method	Principle	Time	Purity	Yield	Cost	Ref.
differential ultracentrifugation	Size and density	>4 h	Medium	Low	expensive equipment	[110,111,112]
gradient density ultracentrifugation	Size and density	>16 h	High	Low	high	[113,114]
Size-exclusion chromatography	Size	Less than 20 min	high	high	Medium to high	[115,116]
Immunoaffinity capture	Specific binding	4–20 h	high	medium	Expensive antibodies functionalization	[109,117]
Ultrafiltration	size and molecular weight	0.5 h	low	medium	medium	[117]
Precipitation	Solubility	0.25–12 h	low	high	low	[118]
microfluidics	Specific binding, size, and density	0.5 h	high	Low to medium	high	[118]

**Table 4:** Summary of LSPR biosensors for the detection of exosomes.

Recognition Element	Specific Target	Developed Biosensor System	Detection Limit		Ref.
			${LOD}_{ori}$	${LOD}_{conv}$ (Exosomes/mL)	
CD63 aptamer	CD63	Colorimetric biosensor where exosome quantification is based on metallization of Au NRs and hybridization chain reaction (HCR)	1.6 × 10^2^ exosomes/mL	1.6 × 10^2^	[157]
anti-CD63	exosome transmembrane protein CD63	LSPR biosensor based on gold nano-ellipsoid arrays integrated with microfluidics	1 ng/mL	7.245 × 10^26^	[17]
HIF-1α- aptamer	HIF-1α	Au NPs with a diameter of 13 nm were functionalized with aptamer. The bond between the ligand and the analyte results in changes in the absorbance intensity.	0.2 ng/L	1.449 × 10^26^	[158]
-	A-549 and SH-SY5Y cells	LSPR biosensor with self-assembly gold nanoislands (SAM-AuNIs)	0.194 µg/mL	1.41 × 10^29^	[159]
locked nucleic acid (LNA)	exo-miR-125b	DNA-assembled advanced plasmonic architecture (DAPA)-based plasmonic biosensor	10.54 aM	6.344 × 10^3^	[27]
anti-hPD-L1 antibody	PD-L1 exosomes	nanoplasmonic sandwich composed of Au@Ag core-shell nanobipyramid (NBP) and AuNR	1.2 × 10^3^ exosomes/μL	1.2 × 10^6^	[16]
CD63 aptamer	CD63	colorimetric biosensors based on Au NBP@MnO_2_ nanostructures	1.35 × 10^2^ exosomes/μL	1.35 × 10^5^	[152]
CA9 Aptamer	Clear-Cell Renal Cancer Exosome	optical microfiber integrated with ${MoSe}_{2}$-supported Au NRs	9.32 exosomes/mL	9.32	[160]

## Data Availability

Not applicable.
